# TI-RADS Diagnostic Performance: Which Algorithm is Superior and How Elastography and 4D Vascularity Improve the Malignancy Risk Assessment

**DOI:** 10.3390/diagnostics10040180

**Published:** 2020-03-26

**Authors:** Andreea Borlea, Florin Borcan, Ioan Sporea, Cristina Adriana Dehelean, Romeo Negrea, Laura Cotoi, Dana Stoian

**Affiliations:** 1 2nd Department of Internal Medicine, Victor Babes University of Medicine and Pharmacy, 300041 Timisoara, Romania; borlea.andreea@umft.ro (A.B.); isporea@umft.ro (I.S.); cotoi.laura@umft.ro (L.C.); stoian.dana@umft.ro (D.S.); 2 Faculty of Pharmacy, Victor Babes University of Medicine and Pharmacy, 300041 Timisoara, Romania; cadehelean@umft.ro; 3 Department of Mathematics, Politehnica University, 300006 Timişoara, Romania; romeo.negrea@upt.ro

**Keywords:** elastography, TI-RADS, 4D Color Doppler, vascularity, malignancy risk, thyroid nodule, stratification

## Abstract

Given the increased prevalence of thyroid nodules in the general population (~50%), the real challenge resides in correctly recognizing the suspicious ones. This study proposes to compare four important Thyroid Imaging and Reporting Data Systems (TI-RADS) and evaluate the contribution of elastography and 4D Color Doppler assessment of vascularity in estimating the risk of malignancy. In the study, 133 nodules with histopathological examination were included. Of these, 35 (26.31%) proved to be malignant. All nodules were classified using the four selected systems and our proposed improved score. The American College of Radiology (ACR) and EU TI-RADS had good sensitivity (94.28%, 97.14%) and NPV (93.33%, 95.83%), but fairly poor specificity (31.81%, 23.46%) and PPV (35.48%, 31.19%), with an accuracy of 42.8% and 45.8%, respectively. Horvath TI-RADS had better accuracy of 66.9% and somewhat improved specificity (62.24%), but poorer sensitivity (80%). Russ’ French TI-RADS includes elastography in the risk assessment strategy. This classification proved superior in all aspects (Se: 91.42%, Sp:82.65%, NPV:96.42%, PPV:65.30%, and Acc of 84.96%). The mean strain ratio (SR) value for malignant lesions was 5.56, while the mean SR value for benign ones was significantly lower, 2.54 (*p* < 0.05). It also correlated well with the response variable: histopathological result (*p* < 0.001). Although, adding 4D vascularity to the French score generated a similar calculated accuracy and from a statistical point of view, the parameter itself proved beneficial for predicting the malignancy risk (*p* < 0.001) and may add important knowledge in uncertain situations. Advanced ultrasound techniques definitely improved the risk estimation and should be used more extensively.

## 1. Introduction

It is acknowledged that the prevalence of thyroid nodules in the general population is rising. Nowadays, easily-accessible advanced imaging techniques bring an important contribution to the increasing number of small asymptomatic nodules that are diagnosed. Nodular thyroid pathology is more commonly associated with female gender and iodine deficiency and its prevalence increases with age. Thyroid malignancy is the actual worry and although its percentage is still small, around 7–15%, it was also reported to have increased over the past few years [1,2].

The most efficient and affordable method to characterize thyroid lesions was demonstrated to be high-resolution ultrasound (US) and its importance in differentiating non-malignant nodules from cancerous ones is crucial. No single sonographic finding has been proven to be predictive of malignancy, but several imaging scores including a number of parameters have been proposed for assessing the risk of cancer. Ideally, a uniform, standardized US classification should be used internationally by all clinicians. Given their important prevalence, many guidelines and papers focused their work on developing the best risk assessment and therapeutic strategies, but their recommendations slightly differ in some respects [3,4].

The use of US-guided fine-needle-aspiration (FNA) cytology has decreased the number of unnecessary thyroid surgeries. The procedure is simple and safe, but overusing it makes it impractical, highlighting the importance of discriminating between suspicious nodules that need to undergo the procedure and low-risk ones. FNA has its limitations: indeterminate cytology, the numerous non-diagnostic results (1.8% up to 23.6%) [5], and the fact that results are operator-dependent and can be influenced by the technique or the nodule’s composition. Only 9–13% of FNA results prove to be malignant, so the relevant criteria for identifying nodules to undergo this procedure could be improved [6,7].

Elastography serves as a valuable non-invasive US tool that measures tissue strain. It is nowadays more and more widely used to better thyroid nodule discrimination. Both real-time elastography (RTE) and shear wave elastography (SWE) proved to have good results in indicating the malignancy risk using increased stiffness as a criterion of suspicion, with superior outcomes for RTE [8,9,10].

The vascularity of the lesion presents an important role in tumor expansion, invasion, and metastasis development [11,12]. However, the Doppler pattern remains controversial and is not included in most of the currently used scoring systems. It is considered extremely dependent on the device and has poor interobserver agreement. Adding 4-dimensional (4D) real-time Color Doppler evaluation as a suspicion criterion may improve the risk-stratification algorithm [13,14,15].

The purpose of the current paper is to evaluate which thyroid imaging scoring system is optimal to use in everyday clinical practice for predicting the malignancy risk and how elastography and 4D vascularity parameters may help improve the stratification models.

## 2. Materials and Methods

A prospective study was conducted in our US unit from January 2017 to May 2018 on patients that presented with solid thyroid nodules suitable for US assessment. The study was performed in accordance with the Ethical Guidelines of the Helsinki Declaration and was approved by the local Ethics Committee. All subjects agreed to the evaluation and gave their written informed consent prior to inclusion.

Patients who had previously undergone thyroid surgery or radiation therapy or who presented with completely cystic nodules were excluded from the study. Scintigraphic evaluation of the thyroid gland was performed in patients with suspicion of hot nodules (TSH below the normal range). Autonomously functioning nodules were found in nine cases and given their very unlikely tendency for malignancy (<1%), these cases were also excluded from the study [16,17,18]. For patients with multinodular goiter, the nodule with the most suspicious US pattern was chosen for inclusion in the study. All patients were sonographically evaluated before the thyroid intervention by an experienced operator (more than 10 years with conventional US, 5 years with RTE). US evaluation included conventional B-mode US, elastography, and 3D Color Doppler assessment. At the end of the inclusion period, only 133 out of the 314 evaluated nodules had a pathology report and were therefore included in the study. The other cases were considered in the final analysis.

### 2.1. Conventional Ultrasound Evaluation and Imaging Scoring Systems

US evaluation, SE, and 3D Doppler evaluations were performed using a Hitachi Preirus (Hitachi Medical Corporation, Tokyo, Japan) machine with a 5–18 MHz linear multifrequency probe and a 5–13 MHz linear 4D volumetric probe. In all cases, a thorough description of US appearance of the nodules was made, including: measurements, composition (solid, mixed, cystic nodules were excluded), shape (taller-than-wide or not), margins (regular, irregular/blurred), echogenicity (hyper-, iso-, hypo-/marked hypoechoic), presence of calcifications (absent, micro- or macro- or rim calcifications), and presence of suspicious lymph nodes.

2D color Doppler characteristics were not included in our risk-assessment strategy. It is well known that malignant tumor growth relies mostly on its vascularity and quantifying it would seem of relevance. Internal vascularity was typically described in malignant lesions while a peripheral vascular pattern was found to be characteristic for benign ones. Nevertheless, poor outcomes have been described for 2D Doppler as a parameter for predicting thyroid malignancy [19,20].

Based on conventional US criteria, the nodules were then classified using four different scoring systems in order to examine which of them evaluated the malignancy risk better after comparing results with the histopathological record, which is considered the gold-standard for diagnosis (Table 1).

Horvath Thyroid Imaging and Reporting Data System (TI-RADS) was one of the selected scores to be compared to, being the first scoring system proposed for thyroid nodules (2009), inspired by the breast lesion scoring system (BI-RADS). The European Thyroid Association EU-TIRADS and American College of Radiology (ACR) TI-RADS are very commonly used in clinical practice and were included in the study for comparison. The French TI-RADS (2016) is the first to include high stiffness as a suspicion feature and for that reason, it was also chosen for comparison in the present study [14,21,22,23,24].

The categories for each calculated TI-RADS system that was included in this research, together with the expected malignancy risk for each category for all scoring systems individually, are detailed in Table 2.

### 2.2. Elastography Measurements

Strain elastography (SE) was performed in all cases using the above-mentioned US equipment. In order to obtain valid images, mild external pressure was applied. A red-green-blue color map is displayed: red is representative of soft tissue, green describes intermediate stiffness (equal strain), and blue represents hard nodules (no strain). The color-map-acquired images were interpreted according to the Asteria criteria: ranging from 1 (entirely elastic lesion) to 4 (entirely stiff) [25].

**Figure 1:**
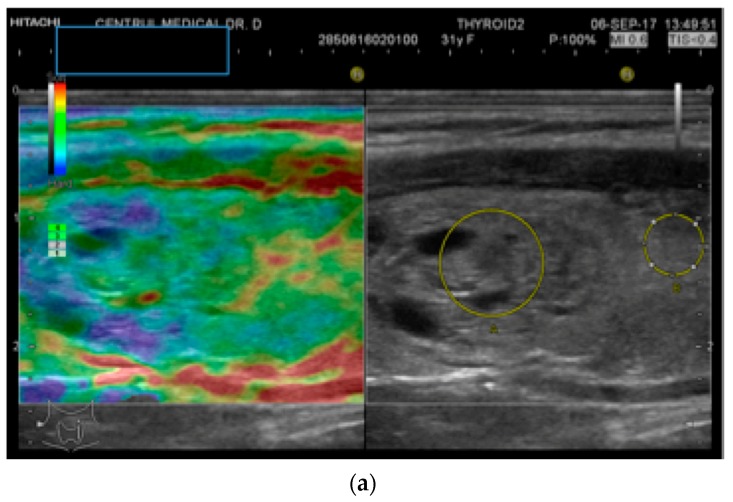

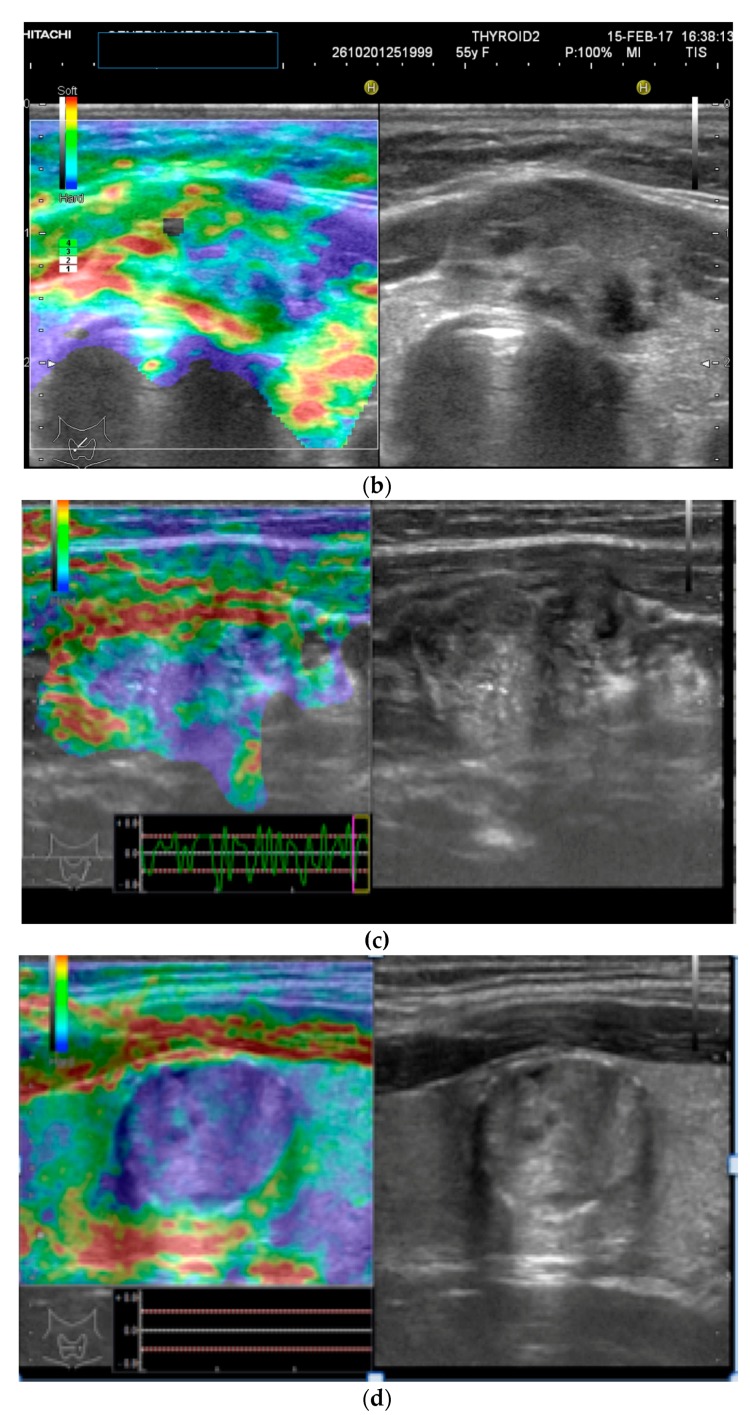
(**a**) Follicular neoplasia with no endothelial invasion, US low risk; (**b**) Noninvasive follicular neoplasia, intermediate US risk; (**c**) Hurthle cell carcinoma—high US risk; (**d**) Follicular adenoma—high US risk.

An objective semi-quantitative determination was also made, supplying a numeric value: the strain ratio (SR). It represents the ratio between two regions-of-interest (ROI) placed at the same depth: the neighboring thyroid parenchyma strain value and the mean strain of the lesion (parenchyma-to-nodule ratio). Longitudinal sections were used in order to have adjacent healthy surrounding thyroid parenchyma on display [26]. See Figure 1.

### 2.3. Volumetric Color Doppler

As previously mentioned, 2D Doppler vascularity alone was found to show poor sensitivity (Se), specificity (Sp), and positive predictive value (PPV) for malignancy. Recent technological developments have helped to improve the acquisition and quality of 3D images using automatic three-dimensional volumetric probes. The nodule is positioned in the center of the image, with 180° rotational scanning inside the volumetric probe. Therefore, a complete evaluation of the entire lesion’s vascularity is achieved, including the vessels spreading from the surrounding parenchyma into the nodule. In cases with rich vascularization, vessels in the periphery and the ones in the center of the nodule can overlap. In this case, thin-sliced images (0.2 mm) in each of the three planes will be observed additionally [15,27].

The integrity of the thyroid capsule (intact/altered) and intranodular vascularization (normal/increased) displayed on 3D Doppler images represent the features of interest when trying to anticipate the risk of malignancy.

**Figure 2:**
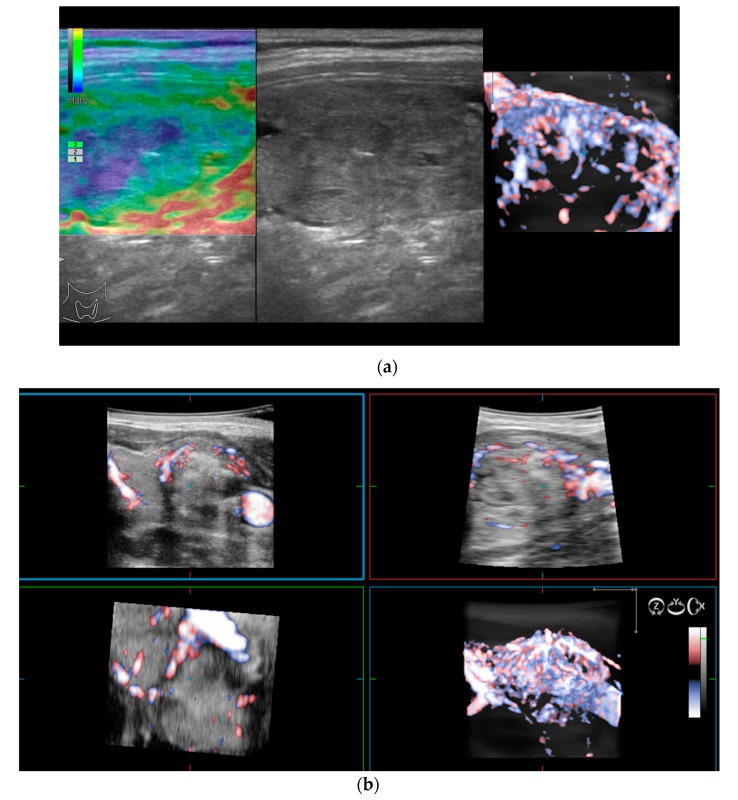
(**a**) Risk upgrade: Strain elastography + 4D vascularisation; (**b**) 4D Color Doppler Risk upgrade: Intermediate -> High.

The 3D images of the scanned region seem to display significantly different patterns for lesions which have a similar aspect in 2D Doppler—as seen in the images below (Figure 2).

### 2.4. Proposed Algorithm

Our proposed algorithm is adapted from the French TI-RADS which includes elastography-documented increased stiffness as a high suspicion parameter. Our algorithm adds 3D Doppler to the evaluation: capsule interruption and increased intranodular vascularization as an additional feature for malignancy suspicion. As for elastography assessment, semi-quantitative elastography (SR > 4) was the parameter considered in our model as predictive of cancerous lesions. Various cut-off values have been proposed in order to clearly differentiate stiff lesions and these values also depend on the equipment that was used. Previous studies in our center, using the same elastography method and the same ultrasound machine, found a threshold value of 4 in this respect [22,28].

The suggested US classification algorithm is comprehensively explained below in Table 3. 

### 2.5. Pathology Examination

The existence of histopathological results (gold standard for diagnosis) was one of the inclusion criteria for the study, thus they were available for all 133 cases. This improved accuracy, but also limited the number of studied nodules. Surgeons performed unilateral lobectomy or total thyroidectomy in all cases. The surgical intervention was recommended due to the presence of suspicious lateral cervical lymph nodes (non-inflammatory pattern) accompanying high-risk nodules in 13 cases. It was required due to indeterminate and positive FNA cytology results (Bethesda III and IV, V and VI, respectively) in 68 cases and due to rapid growth in 12 cases. The remaining 33 cases opted for surgical therapy due to compression-only effects, multinodularity associated with compression, or cosmetic reasons.

### 2.6. Statistical Analysis

Data were collected and analyzed using R statistical software. Se, Sp, PPV, negative predictive values (NPV), and accuracy (Acc) were calculated for each score, using pathology reports as the diagnosis gold standard. Given that the response variable is a categorical (binary) random variable, the generalized linear regression method, in fact, binomial (logistic) generalized linear regression, was used for analysis, i.e., a multiple linear relation between the logistic function of the probability of the occurrence of the response variable and the linear combination of predictor variables. Statistical deviance, Akaike Information Criterion (AIC), and Fisher scoring were used in order to compare the proposed models and how 3D Doppler improves the score.

## 3. Results

133 nodules (nodules with most suspicious features or rapid-growth) were finally analyzed in 133 patients, 96 females and 37 males, with a mean age of 45.3. The pathology reports revealed benign findings in 98 cases and thyroid cancer in 35 cases (26.31%): papillary carcinoma in 22 cases (62.85%), follicular carcinoma in 9 cases (25.71%), Hurthle-cell carcinoma in one case (2.85%), medullary carcinoma in one patient (2.85%), and 2 cases with borderline follicular cell tumors (5.71%). This last mentioned category was included in the malignant group due to its unclear risk and progression [29]. The mean SR value for malignant lesions was 5.56, while the mean SR value for benign ones was significantly lower (2.54, *p* < 0.05), See Table 4.

SR seems to be the most constantly described suspicious feature in histologically confirmed cancer cases. As for the benign group, increased stiffness was found in some Hurthle adenomas, granulomatous lesions, and probably some long-standing fibrous nodules.

All 133 nodules were classified using the four selected TI-RADS models and a fifth algorithm that we designed, including 4D vascularity evaluation. The EU-TIRADS and ACR TI-RADS generated a great number of high risk lesions (categories 4 and 5) and, therefore, had a poor calculated risk for this group. It did show a low calculated risk for low-risk lesions, but the number in these categories was too small. This may have also been influenced by the inclusion of only solid nodules in the study. Our team found Horvath TI-RADS more difficult to use and less user-friendly. One malignant lesion was classified as category 2, while the risk for category 5 was calculated to be around 55%.

The French TI-RADS seemed to sort lesions more appropriately according to both conventional US characteristics algorithm and the extra-parameter: stiffness. Further, 88.1% of the TIRADS 5 lesions proved to be malignant. In our group, no malignancy was found in categories 2 and 3 of this classification. Our improved algorithm added 4D vascularization to the previous classification. A slight improvement was noted for the calculated risk. These findings are presented in detail in Table 5.

The calculated diagnostic values are displayed comparatively in Table 6. The ACR and EU TI-RADS had similar results, with good sensitivity and NPV, but very poor specificity and PPV, with an accuracy of 42.8% and 45.8%, respectively. Horvath TI-RADS had an accuracy of 66.9% and a slightly improved PPV and specificity. Russ’ French TI-RADS proved superior in all aspects, with a diagnostic accuracy of 84.96%. Adding a 4D vascularity feature did not seem to improve accuracy and somewhat decreased PPV and specificity.

In order to better evaluate the contribution of a three-dimensional Doppler assessment, as well as all other included parameters, some correlations were calculated and compared to histopathological findings, which is the golden standard for diagnosis.

As shown in Table 7, some good correlations were observed between the predictor variables: SR, 3D Doppler and French TIRADS score (0.5053765, 0.6506053, 0.5531696) and the response variable (histopathological exam). The first remark was that 3D vascularity describes good correspondence with histopathological findings.

Binomial logistic generalized linear regression was used to analyze the relation between the probability of the response variable and the predictor variables.

First, we analyzed the relationship between the response variable (histopathological result) and the predictor variables (Taller-than-wide, calcification, SR, and hypoechogenicity) using the R statistical software. From a statistical point of view (*t*-test), only SR (*p* < 0.001) was a very good predictor; taller-than-wide proved somewhat good for predicting malignancy (*p* = 0.12), but the other parameters did not show good predictions (*p* = 0.292506, *p* = 0.716407, *p* = 0.794967). Next, 3D Doppler evaluation was added as a predictor to be analyzed and proved to be efficient (with respect to the *p*-value of the *t*-test= 0.0002). The generalized relation between the improved score (including 3D Doppler) as a predictor variable and response variable (histopathological exam) was very good (*p* < 0.001), and a similar result was obtained when classic French TI-RADS was compared to the standard for diagnosis (*p* < 0.001), hence the relationship is strong and important.

From the above-mentioned data, we may conclude that the additional predictor variable 3D vascularity, which was proposed as an extra parameter to Russ’ Classification, is statistically important.

French TI-RADS had the best diagnostic quality out of the included algorithms for comparison. This is why the proposed additional parameter was added to this specific score. When comparing the two models (with and without 3D Doppler), statistical deviance, AIC, and Fisher Scoring were used.

The statistical deviance measures the goodness-of-fit of a generalized linear model. It showed a somewhat better outcome for the French TI-RADS (81.41) compared to our score (96.86), and the higher the deviance value, the poorer the model fit.

AIC was used to compare the goodness-of-fit of the two models. It is a measure of a model’s flexibility, with the model showing a lower AIC considered to be better. It was applied in order to determine (penalize) if the evaluated models were too complicated or include irrelevant predictors. In our case, AIC showed a value of 85.416 for the first model and 100.86 for the second, which is in favor of the simple French model.

Fisher scoring aim represents the estimation of the parameters; it stops when no further improvement can be made. In our case, it was 5 and 6, respectively, showing that 3D may actually improve the algorithm.

From the statistical analysis, we can confirm that SR is an important predictor for malignancy, independently but also as part of the combined US evaluation algorithm. Although the proposed score did not seem to greatly improve the diagnostic performance (Se, Sp, PPV, NPV, Acc), 3D Doppler assessment is important for US evaluation in all cases. On the other hand, the statistical parameters used for the generalized regression suggest that the simple French TI-RADS model is better than the model including vascularization. Therefore, we can conclude that elastography and real-time Doppler evaluation (4D) both bring an important improvement compared to classic, conventional B-mode US, with a substantially more valuable contribution for elastography compared to 4D.

## 4. Discussion

Conventional ultrasound parameters are quite well studied. Although individually they do not display a good predictive value for the malignancy risk, the different proposed algorithms comprising their association have better outcomes [30,31]. The best recipe in this respect has yet to be found, but intensive clinical research is aimed at improving the existing ones. Instead of developing a standardized, universal evaluation of thyroid nodules, this has led to inconsistent, widely varying evaluation and classification of thyroid lesions. Fine-needle-aspiration is overused and has its limitations, underlining the need for an essential improvement in the US selection of nodules. The importance of lesion size stems from its inverse relationship with prognosis [31,32,33].

Given the important number of various ultrasound assessment scores, the present study could not include all of them. The ones that are widely used in our region were included (the EU- and ACR TI-RADS), together with the first US classification model by E. Horvath (2009). The French TI-RADS developed by Russ et al. was the first model to include elastography in the risk assessment, adding great value to the classic scores. It was also included in the present study for estimating the contribution of stiffness as a risk parameter [14,21,23,24].

It is worth mentioning that each stratification system was developed for its local population. Factors like iodine sufficiency and genetic variations may generate differences in the development of thyroid lesions, but there is not enough evidence of variant malignancy patterns for different populations. The present study was performed in a Caucasian European population from the Western part of Romania who were deemed iodine sufficient [34].

Firstly, sensitivity, specificity, PPV, NPV, and accuracy of the four classifications were determined and compared. The first proposed model by Horvath et al. was inspired by the breast evaluation score (BI-RADS) and contributed valuable progress for monitoring and managing thyroid lesions. It was later described as being too complicated for clinical use [35]. Our team also found it difficult to apply, deeming it puzzling and time-consuming. It is worth mentioning that our findings in terms of diagnostic performance differ from the proposed ones. The highly-suspicious category has an expected risk of more than 80%, while the present study found a calculated risk of only 55.17%.

The EU-TIRADS was probably the most time-efficient and simple-structured algorithm. The calculated risk for the study group was similar to the expected risk, with the mention that given intervals are noticeably wide, especially for the highly suspicious category: 26%–87%. Moreover, the calculated degrees of prediction show poor results for some of the high-risk features taken individually. Although good sensitivity (97.14%) was described for this model in the study group, classifying nodules that present any one of these described features as high-suspicious lesions may justify the many false negatives, with poor specificity (23.46%) and NPV (31.19%). No malignancy was found in EU-TIRADS category 2 and only one in category 3, where FNA is recommended only for lesions greater than 20 mm.

ACR TI-RADS proved to have a similar diagnostic quality, with a minor improvement in terms of specificity and NPV, but still too many false negatives, resulting in unnecessary FNAs. Its calculated diagnostic accuracy of 25.86% is still unsatisfactory and could be improved. Although in the beginning it may take longer, compared to EU-TIRADS, to check all parameters and determine the ACR category, it prevents the observer from overlooking any features.

A recent meta-analysis including 12 studies and 18,750 nodules in an adult population made a head-to-head comparison of the ability of ACR, EU, K-TIRADS, American Thyroid Association (ATA), and American Association of Clinical Endocrinologists (AACE) risk stratification systems. The authors found a better performance for ACR TI-RADS in selecting nodules for FNA, due to its superior relative likelihood ratio for positive outcomes [36].

Given the important value that elastography assessment brings to nodular thyroid pathology evaluation, French TI-RADS, which includes elastography in the risk stratification, has been considered in the performance comparison. High stiffness represents the extra feature taken into consideration for malignancy risk stratification. Elastography provides a non-invasive assessment of tissue stiffness. Both RTE and SWE add relevant information for classifying nodules, as specified in the European Federation of Societies for Ultrasound in Medicine and Biology (EFSUMB) guidelines, but SE proved superior. It should not be used alone, nor should it replace grey-scale US, but should be used complementary to it [10,14,37,38]. Unfortunately, this powerful tool is still underrated and not sufficiently used for thyroid evaluation. Considered as an independent predictor of malignancy, elastography showed the best sensitivity-specificity combination in comparison to the other US parameters [39].

The assessment in this study included both qualitative (color-map) and quantitative (SR) elastography evaluation, but SR was the additional parameter to be used in risk stratification. SR >4 was found in 80% of the investigated malignant nodules and in only 12% of the benign ones. Of course, different values are described for diagnostic performance in the literature, depending on the equipment and type of elastography that was used [40,41]. The authors of the French TI-RADS described specificity, NPV, and accuracy of 44.7%, 99.8%, and 48.3%, respectively [21]. In another study, they describe a comparative sensitivity of 93.2%, 41.9%, and 96.7% for conventional B-mode evaluation, elastography, and a model combining the two, underlining the importance of an overall interpretation instead of considering them individually [42]. Stoian et al. used SR quantitative strain elastography (SR) as the additional parameter for measuring stiffness, with better values and an overall accuracy of 95.97% [22].

Another important question is raised by the presence of indeterminate results. Here, also, elastography proved to be helpful with decision-making, whether to watch and wait or send the patient to surgery [28]. Molecular markers may be helpful in these situations, with important specificity and PPV, but low sensitivity and NPV; there are no clear recommendations regarding their use and their cost-efficiency is debatable [43,44]. Follicular variant papillary thyroid cancer (PTC) often lacks in US features of high suspicion and may escape diagnosis [45]. Elastography has its limitations: follicular thyroid cancer (FTC) usually presents low stiffness, while rim calcifications may increase stiffness; in predominantly cystic nodules, the stiffness of the solid part may be difficult to measure and soft areas may be present inside necrosis [46,47].

A combined evaluation including advanced ultrasound applications is proposed in this study. Vascular pattern of a nodule seems of great value, but classic Doppler evaluation showed poor results [20]. Besides qualitative and quantitative strain elastography, volumetric Doppler evaluation was also used. 3D Doppler documentation of an altered thyroid capsule or increased intranodular vascularization were considered as predictive of malignancy and were included in the proposed stratification model. To our knowledge, few studies have used this combined strategy. Volumetric Doppler evaluation did prove to be a good predictor of malignancy (*p* = 0.002). As for comparing the French score (conventional US + SR) versus the improved algorithm (conventional + SR + 4D), the diagnostic quality did not seem to improve greatly with the addition of 4D parameter, showing a calculated sensitivity of 91.42% versus 94.28%, specificity of 82.65% versus 75.51%, PPV of 65.30% versus 57.89%, NPV of 96.42% versus 97.36%, and accuracy of 84.96% versus 80.45%.

Both parameters proved legitimately valuable as additional high-risk features, but SR was by far the best evaluator, both individually and as part of the proposed classification.

One important limitation of the study is represented by the narrow sample size (133 nodules, out of which 35 were malignant). Given the innovative approach, the scarce data available on 4D Doppler evaluation of thyroid lesions, and its promising potential as an additional parameter in assessing the risk of malignancy, proved by the present paper, we consider that the study still adds value to the current knowledge. It certainly claims a need to extend the research to a larger population with broader pathology.

As for future applications, thyroid contrast-enhanced US (CEUS) has great potential, in addition to US evaluation of thyroid lesions. It is widely used in describing liver focal lesions, reducing the number of unnecessary biopsies. Little research has been done using contrast US for thyroid lesions. Hypoenhancement and heterogeneity describe malignant lesions, while homogeneity and the presence of an enhanced peripheral ring indicate a benign nodule. However, more research is necessary in this promising field [48,49,50].

## 5. Conclusions

All Imaging Reporting Systems have good detection for the high-risk nodules, each of them with their particular strengths and weaknesses. It is important for clinicians to select the one that they thoroughly understand and can easily use in everyday clinical practice in order to help them decide what to do next with a nodule: follow or perform FNA. There is good evidence that both strain and shear-wave elastography greatly improve the US assessment, with better results for the first one. Unfortunately, stiffness measurements are not yet largely used in thyroid US evaluation, but they are becoming more and more popular. Determining the stiffness of a lesion may be the most useful parameter that we can add to the current conventional proposed scores. It significantly improved the stratification algorithm in this study. High stiffness was found in 80% of malignant lesions and only 12% of benign ones. These findings support the considerable value that this additional investigation brings, suggesting that it should be included in the evaluation, if available.

From the statistical analysis, 3D Doppler tested as a very good predictor of malignancy. The improved score after including this parameter was also very good. In terms of calculated diagnostic quality, it showed similar accuracy to the French score, decreasing the percentage of false negatives (failure to detect cancer), while also slightly increasing the false positive percentage (falsely diagnosed cancers). In this regard, it is important to underline that the extra parameter should not downgrade the risk, rather it should only upgrade the risk when the integrity of the thyroid capsule is altered and/or increased intranodular vascularization is described by volumetric Doppler evaluation. The modest sample size, the distribution of malignancy in the study group, and the small number of certain types of thyroid cancer limits the power of the study. A definite conclusion concerning volumetric Doppler’s usefulness in detecting malignant lesions cannot be drawn yet. The vascularity assessment using this technique can perhaps be improved. Nevertheless, the study brings relevant observations regarding its promising benefit in the evaluation of thyroid nodules and demonstrates the need to extend the research.

Advanced ultrasound techniques definitely seem promising in thyroid nodular pathology, adding valuable information to the gray-scale evaluation.

## Figures and Tables

**Table 1:** Comparative US Classification of Nodules used in the study: European Thyroid Imaging and Reporting Data System (TI-RADS) versus American College of Radiology (ACR) TI-RADS (2016) versus Horvath TI-RADS (2009) versus French TI-RADS.

Category	EU-TIRADS	ACR TIRADS *	Horvath TIRADS	French TIRADS (Includes Elastography)
1	No nodules.	Score = 0	No nodules.	No nodules.
2	Cyst/Spongiform.	Score = 2	Colloid/spongiform/Mixed isoechoic.	Cyst/Isolated macrocalcification/Spongiform
3	Oval, smooth margins, iso-/hyperechoic, no suspicious feature.	Score = 3	Hashimoto pseudo-nodule.	Oval, regular margins, iso/hyperechoic
4	Oval, smooth margins, mild hypoechoic, no suspicious feature.	Score = 4–6	4A: Hyper, iso, or hypoechoic nodule + thin capsule Hypoechoic, ill-defined borders, no calcifications Hypervascularized, nodule with thick capsule and calcifications.	4A: Oval, regular margins, mild hypoechoic
			4B: Hypoechoic, irregular shape and margins, penetrating vessels ±calcifications	4B: High suspicion features (1–2) Taller-than-wide Irregular margins Marked hypoechoic Microcalcifications Stiff on elastography
5	Suspicious features (min 1): -irregular shape -irregular margins -microcalcifications -marked hypoechoic	Score ≥ 7	Iso/hypoechoic, nonencapsulated multiple peripheral microcalcifications hypervascular Nonencapsulated, isoechoic mixed, hypervascular ± calcifications	High suspicion features (3–5) and/or Lymph node metastasis

* For the ACR TI-RADS, a cumulative score from five categories of ultrasound findings is determined (composition, echogenicity, shape, margins and presence of echogenic foci).

**Table 2:** Comparative US Classification of Nodules: Categories for each scoring system with the expected malignancy risk for each category.

	EU-TIRADS		ACR-TIRADS		Horvath TIRADS		French TIRADS (Includes Elastography)	
Normal gland	1	-			1	-	1	-
Benign	2	~0%	1	0.3%	2	0%	2	0%
Not suspicious			2	1.5%	3	<5%	3	0.25%
Mildly suspicious	3	2% to 4%	3	4.8%	4A	5–10%	4A	6%
Moderately suspicious	4	6–17%	4	9.1%	4B	10–80%	4B	69%
Highly suspicious	5	26–87%	5	35%	5	>80%	5	~100%
Biopsy-proven malignancy					6	100%		

**Table 3:** Proposed algorithm for US classification of thyroid nodules, including Volumetric Color Doppler evaluation. Adapted from French TI-RADS, Russ, 2016 [21].

Category	French TIRADS + 4D Color Doppler
1	No nodules
2	Cyst/Isolated macrocalcification/Spongiform
3	Oval, regular margins, iso/hyperechoic
4A	Oval, regular margins, mild hypoechoic
4B	High suspicion features (1 or 2): Taller-than-wide Irregular margins Marked hypoechoic Microcalcifications Stiff on elastography *(quantitative SE: SR > 4)* Increased intranodular vascularity/interrupted capsule (3D CD assessment)
5	High suspicion features (3–6) and/or Lymph node metastasis

**Table 4:** Prevalence of suspicious features in benign and malignant groups.

US Characteristic	Benign	Malignant
Blurred margins	28 (28.57%)	13 (37%)
Microcalcification	9 (9.1%)	11 (31.4%)
Marked Hypoechoic	3 (3.06%)	9 (25.7%)
Taller-than-wide	15 (15.3%)	15 (42.8%)
SR (>4)	12 (12.24%)	28 (80%)
4D: increased intranodular Vascularity/interrupted capsule	14 (14.28%)	23 (65%)

**Table 5:** Comparison of TI-RADS scores: Histopathological findings in the study group for each category and calculated malignancy risk for each model.

	Total	Benign	Malignant	Calculated Risk
**EU TI-RADS**				
2	6	6	0	0%
3	18	17	1	5.55%
4	68	56	12	17.6%
5	41	19	22	53%
**ACR TI-RADS**				
1	5	5	0	0%
2	25	23	2	8%
3	0	0	0	-
4	64	48	16	25%
5	39	22	17	43.58%
**Horvath TI-RADS**				
2	19	18	1	5.26%
3	5	5	0	0%
4A	44	38	6	13.63%
4B	36	24	12	33.33%
5	29	13	16	55.17%
**French TI-RADS**				
2	6	6	0	0%
3	19	19	0	0%
4A	59	56	3	5.08%
4B	27	13	14	51.85%
5	22	4	18	81.8%
**French TI-RADS + 4D CD**				
2	6	6	0	0%
3	19	19	0	0%
4A	51	49	2	3.92%
4B	32	20	12	37.5%
5	25	4	21	84%

**Table 6:** Compared Diagnostic Quality: sensitivity (Se), specificity (Sp), positive predictive value (PPV), negative predictive value (NPV), and accuracy for the proposed TI-RADS scores.

	Se (%)	Sp (%)	PPV (%)	NPV (%)	Accuracy (%)
EU TI-RADS	97.14	23.46	31.19	95.83	42.85
ACR TI-RADS	94.28	31.81	35.48	93.33	45.86
Horvath TI-RADS	80	62.24	43.07	89.70	66.91
French TI-RADS	91.42	82.65	65.30	96.42	84.96
French TI-RADS + 4D	94.28	75.51	57.89	97.36	80.45

**Table 7:** Correlation matrix of the variables.

**Taller-than-Wide**	1						
**Marked Hypo- Echogenicity**	0.3323610	1					
**Micro- Calcification**	0.1252548	0.4548725	1				
**3D Doppler Pattern**	0.2269876	0.1558775	0.1612967	1			
**Elastography (SR)**	0.2737142	0.4801859	0.4121376	0.3612075	1		
**French TI-RADS**	0.3885093	0.3871947	0.3494646	0.4956183	0.6428621	1	
**Histopatho- Logical Exam**	0.2902722	0.3481694	0.2740459	0.5053765	0.6506053	0.5531696	1
	**Taller-than-Wide**	**Marked Hypo- Echogenicity**	**Micro-Calcification**	**3D Doppler Pattern**	**Elastography (SR)**	**French TI-RADS**	**Histopatho-Logical Exam**

## References

[1] Moon J.H., Hyun M.K., Lee J.Y., Shim J.I., Kim T.H., Choi H.S., Ahn H.Y., Kim K.W., Park D.J., Park Y.J. (2018). Prevalence of thyroid nodules and their associated clinical parameters: A large-scale, multicenter-based health checkup study. Korean J. Intern. Med..

[2] Paschou S.A., Vryonidou A., Goulis D.G. (2017). Thyroid nodules: Alpha guide to assessment, treatment and follow-up. Maturitas.

[3] Kwong N., Medici M., Angell T.E., Liu X., Marqusee E., Cibas E.S., Krane J.F., Barletta J.A., Kim M.I., Larsen P.R. (2015). The influence of patient age on thyroid nodule formation, multinodularity, and thyroid cancer risk. J. Clin. Endocrinol. Metab..

[4] Cantisani V., David E., Grazhdani H., Rubini A., Radzina M., Dietrich C.F., Durante C., Lamartina L., Grani G., Valeria A. (2019). Prospective Evaluation of Semiquantitative Strain Ratio and Quantitative 2D Ultrasound Shear Wave Elastography (SWE) in Association with TIRADS Classification for Thyroid Nodule Characterization. Ultraschall der Medizin.

[5] Bongiovanni M., Spitale A., Faquin W.C., Mazzucchelli L., Baloch Z.W. (2012). The Bethesda System for Reporting Thyroid Cytopathology: A Meta-Analysis. Acta Cytol..

[6] Krishnappa P., Ramakrishnappa S., Kulkarni M.H. (2013). Comparison of free hand versus ultrasound-guided fine needle aspiration of thyroid with histopathological correlation. J. Environ. Pathol. Toxicol. Oncol..

[7] Kwak J.Y. (2013). Indications for fine needle aspiration in thyroid nodules. Endocrinol. Metab..

[8] Hu X., Liu Y., Qian L. (2017). Diagnostic potential of real-time elastography (RTE) and shear wave elastography (SWE) to differentiate benign and malignant thyroid nodules: A systematic review and meta-analysis. Medicine.

[9] Tian W., Hao S., Gao B., Jiang Y., Zhang X., Zhang S., Guo L., Yan J., Luo D. (2016). Comparing the Diagnostic Accuracy of RTE and SWE in Differentiating Malignant Thyroid Nodules from Benign Ones: A Meta-Analysis. Cell. Physiol. Biochem..

[10] Bamber J., Cosgrove D., Dietrich C.F., Fromageau J., Bojunga J., Calliada F., Cantisani V., Correas J.M., D’Onofrio M., Drakonaki E.E. (2013). EFSUMB guidelines and recommendations on the clinical use of ultrasound elastography. Part 1: Basic principles and technology. Ultraschall Med..

[11] Rajabi S., Dehghan M.H., Dastmalchi R., Jalali Mashayekhi F., Salami S., Hedayati M. (2019). The roles and role-players in thyroid cancer angiogenesis. Endocr. J..

[12] Tasoulas J., Tsourouflis G., Theocharis S. (2018). Neovascularization: An attractive but tricky target in thyroid cancer. Expert Opin. Ther. Targets..

[13] Moon H.J., Kwak J.Y., Kim M.J., Son E.J., Kim E.K. (2010). Can vascularity at power Doppler US help predict thyroid malignancy?. Radiology.

[14] Russ G., Bonnema S.J., Erdogan M.F., Durante C., Ngu R., Leenhardt L. (2017). European Thyroid Association Guidelines for Ultrasound Malignancy Risk Stratification of Thyroid Nodules in Adults: The EU-TIRADS. Eur. Thyroid J..

[15] Stoian D., Ivan V., Sporea I., Florian V., Mozos I., Navolan D., Nemescu D. (2020). Advanced Ultrasound Application—Impact on Presurgical Risk Stratification of the Thyroid Nodules. Ther. Clin. Risk Manag..

[16] Ulusoy B. (2015). The Management of Thyroid Nodules. Turk. Arch. Otorhinolaryngol..

[17] Bomeli S.R., LeBeau S.O., Ferris R.L. (2010). Evaluation of a thyroid nodule. Otolaryngol. Clin. N. Am..

[18] Giovanella L., Treglia G., Trimboli P. (2018). Thyroid imaging. Encyclopedia of Endocrine Diseases.

[19] Caresio C., Caballo M., Deandrea M., Garberoglio R., Mormile A., Rossetto R., Limone P., Molinari F. (2018). Quantitative analysis of thyroid tumors vascularity: A comparison between 3-D contrast-enhanced ultrasound and 3-D Power Doppler on benign and malignant thyroid nodules. Med. Phys..

[20] Lyshchik A., Moses R., Barnes S.L., Higashi T., Asato R., Miga M.I., Gore J.C., Fleischer A.C. (2007). Quantitative analysis of tumor vascularity in benign and malignant solid thyroid nodules. J. Ultrasound Med..

[21] Russ G. (2016). Risk stratification of thyroid nodules on ultrasonography with the French TI-RADS: Description and reflections. Ultrason (Seoul, Korea).

[22] Stoian D., Timar B., Derban M., Pantea S., Varcus F., Craina M., Craciunescu M. (2015). Thyroid Imaging Reporting and Data System (TI-RADS): The impact of quantitative strain elastography for better stratification of cancer risks. Med. Ultrason..

[23] Horvath E., Majlis S., Rossi R., Franco C., Niedmann J.P., Castro A., Dominguez M. (2009). An ultrasonogram reporting system for thyroid nodules stratifying cancer risk for clinical management. J. Clin. Endocrinol. Metab..

[24] Tessler F.N., Middleton W.D., Grant E.G., Hoang J.K., Berland L.L., Teefey S.A., Cronan J.J., Beland M.D., Desser T.S., Frates M.C. (2017). ACR Thyroid Imaging, Reporting and Data System (TI-RADS): White Paper of the ACR TI-RADS Committee. J. Am. Coll. Radiol..

[25] Sigrist R.M.S., Liau J., Kaffas AEl Chammas M.C., Willmann J.K. (2017). Ultrasound Elastography: Review of Techniques and Clinical Applications. Theranostics.

[26] Aydin R., Elmali M., Polat A.V., Danaci M., Akpolat I. (2014). Comparison of muscle-to-nodule and parenchyma-to-nodule strain ratios in the differentiation of benign and malignant thyroid nodules: Which one should we use?. Eur. J. Radiol..

[27] Slapa R.Z., Jakubowski W.S., Slowinska-Srzednicka J., Szopinski K.T. (2011). Advantages and disadvantages of 3D ultrasound of thyroid nodules including thin slice volume rendering. Thyroid Res..

[28] Stoian D., Borcan F., Petre I., Mozos I., Varcus F., Ivan V., Cioca A., Apostol A., Dehelean C.A. (2019). Strain elastography as a valuable diagnosis tool in intermediate cytology (Bethesda III) thyroid nodules. Diagnostics.

[29] Kakudo K. (2018). How to handle borderline/precursor thyroid tumors in management of patients with thyroid nodules. Gland Surg..

[30] Alexander E.K., Pearce E.N., Brent G.A., Brown R.S., Chen H., Dosiou C., Grobman W.A., Laurberg P., Lazarus J.H., Mandel S.J. (2017). 2017 Guidelines of the American Thyroid Association for the Diagnosis and Management of Thyroid Disease During Pregnancy and the Postpartum. Thyroid.

[31] Hoang J.K., Middleton W.D., Farjat A.E., Langer J.E., Reading C.C., Teefey S.A., Abinanti N., Boschini F.J., Bronner A.J., Dahiya N. (2018). Reduction in Thyroid Nodule Biopsies and Improved Accuracy with American College of Radiology Thyroid Imaging Reporting and Data System. Radiology.

[32] Machens A., Holzhausen H.J., Dralle H. (2005). The prognostic value of primary tumor size in papillary and follicular thyroid carcinoma: A comparative analysis. Cancer.

[33] Jabar A.S.S., Koteshwara P., Andrade J. (2019). Diagnostic reliability of the Thyroid Imaging Reporting and Data System (TI-RADS) in routine practice. Pol. J. Radiol..

[34] Gaengler S., Andrianou X.D., Piciu A., Charisiadis P., Zira C., Aristidou K., Piciu D., Makris K.C. (2017). Iodine status and thyroid nodules in females: A comparison of Cyprus and Romania. Public Health.

[35] Park J.-Y., Lee H.J., Jang H.W., Kim H.K., Yi J.H., Lee W., Kim S.H. (2009). A proposal for a thyroid imaging reporting and data system for ultrasound features of thyroid carcinoma. Thyroid.

[36] Castellana M., Castellana C., Treglia G., Giorgino F., Giovanella L., Russ G., Trimboli P. (2019). Performance of five ultrasound risk stratification systems in selecting thyroid nodules for FNA. A meta-analysis. J. Clin. Endocrinol. Metab..

[37] Cosgrove D., Barr R., Bojunga J., Cantisani V., Chammas M.C., Dighe M., Vinayak S., Xu J.M., Dietrich C.F. (2017). WFUMB Guidelines and Recommendations on the Clinical Use of Ultrasound Elastography: Part 4. Thyroid. Ultrasound Med. Biol..

[38] Park A.Y., Son E.J., Han K., Youk J.H., Kim J.-A., Park C.S. (2015). Shear wave elastography of thyroid nodules for the prediction of malignancy in a large scale study. Eur. J. Radiol..

[39] Bojunga J., Herrmann E., Meyer G., Weber S., Zeuzem S., Friedrich-Rust M. (2010). Real-time elastography for the differentiation of benign and malignant thyroid nodules: A meta-analysis. Thyroid.

[40] Xing P., Wu L., Zhang C., Li S., Liu C., Wu C. (2011). Differentiation of Benign From Malignant Thyroid Lesions. J. Ultrasound Med..

[41] Dudea S.M., Botar-Jid C. (2015). Ultrasound elastography in thyroid disease. Med. Ultrason..

[42] Russ G., Royer B., Bigorgne C., Rouxel A., Bienvenu-Perrard M., Leenhardt L. (2013). Prospective evaluation of thyroid imaging reporting and data system on 4550 nodules with and without elastography. Eur. J. Endocrinol..

[43] Sahli Z.T., Smith P.W., Umbricht C.B., Zeiger M.A. (2018). Preoperative Molecular Markers in Thyroid Nodules. Front. Endocrinol. (Lausanne).

[44] Ward L.S., Kloos R.T. (2013). Molecular markers in the diagnosis of thyroid nodules. Arq. Bras. Endocrinol. Metabol..

[45] Chaigneau E., Russ G., Royer B., Bigorgne C., Bienvenu-Perrard M., Rouxel A., Leenhardt L., Belin L., Buffet C. (2018). TIRADS score is of limited clinical value for risk stratification of indeterminate cytological results. Eur. J. Endocrinol..

[46] Hong Y., Wu Y., Luo Z., Wu N., Liu X. (2012). Impact of nodular size on the predictive values of gray-scale, color-Doppler ultrasound, and sonoelastography for assessment of thyroid nodules. J. Zhejiang Univ. Sci. B.

[47] Oliver C., Vaillant-Lombard J., Albarel F., Berbis J., Veyrieres J.B., Sebag F., Petit P. (2011). What is the contribution of elastography to thyroid nodules evaluation?. Ann. Endocrinol. (Paris).

[48] Zhan J., Ding H. (2018). Application of contrast-enhanced ultrasound for evaluation of thyroid nodules. Ultrasonography (Seoul, Korea).

[49] Nolsøe C.P., Lorentzen T. (2016). International guidelines for contrast-enhanced ultrasonography: Ultrasound imaging in the new millennium. Ultrasonography (Seoul, Korea).

[50] Sun B., Lang L., Zhu X., Jiang F., Hong Y., He L. (2015). Accuracy of contrast-enhanced ultrasound in the identification of thyroid nodules: A meta-analysis. Int. J. Clin. Exp. Med..

